# Iatrogenic left vertebral artery pseudoaneurysm treated with a covered stent

**DOI:** 10.1259/bjrcr.20190051

**Published:** 2020-09-29

**Authors:** Miguel Ángel Carrillo-Martínez, German Alfonso Garza García, Juan Manuel Leal Jacinto

**Affiliations:** 1 Interventional Radiology Department, Hospital San José Tec Salud, Monterrey, Mexico; 2 Tecnologico de Monterrey, Escuela de Medicina y Ciencias de la Salud, Monterrey, Mexico

## Abstract

Vertebral artery pseudoaneurysm usually occurs after major trauma, but it can arise spontaneously after trivial injury. Clinical manifestations are often related to alterations in the posterior brain circulation. CT and angiography are usually the diagnostic methods of choice. We present a case of a pseudoaneurysm of the left vertebral artery caused by a lesion during a cervical spine surgery and treated with endovascular approach with a covered stent.

## Clinical presentation

A 57-year-old male with no significant past medical history presented with a 3 year history of intermittent right arm pain, hypoesthesia in the right thumb and index fingers that was progressively disabling. He was admitted to the hospital for surgical treatment of severe degenerative changes and cervical disc hernia (C4–C5 and C5–C6) demonstrated by MRI.

During the surgery, abnormal bleeding presented in the left side of the surgical field during the traction maneuver trying to position disc prosthesis in C4-C5. The bleeding was controlled with compression and electrocoagulation. Arterial lesion was suspected and the interventional radiology team was consulted.

## Investigation/imaging findings

**Figure 1.**
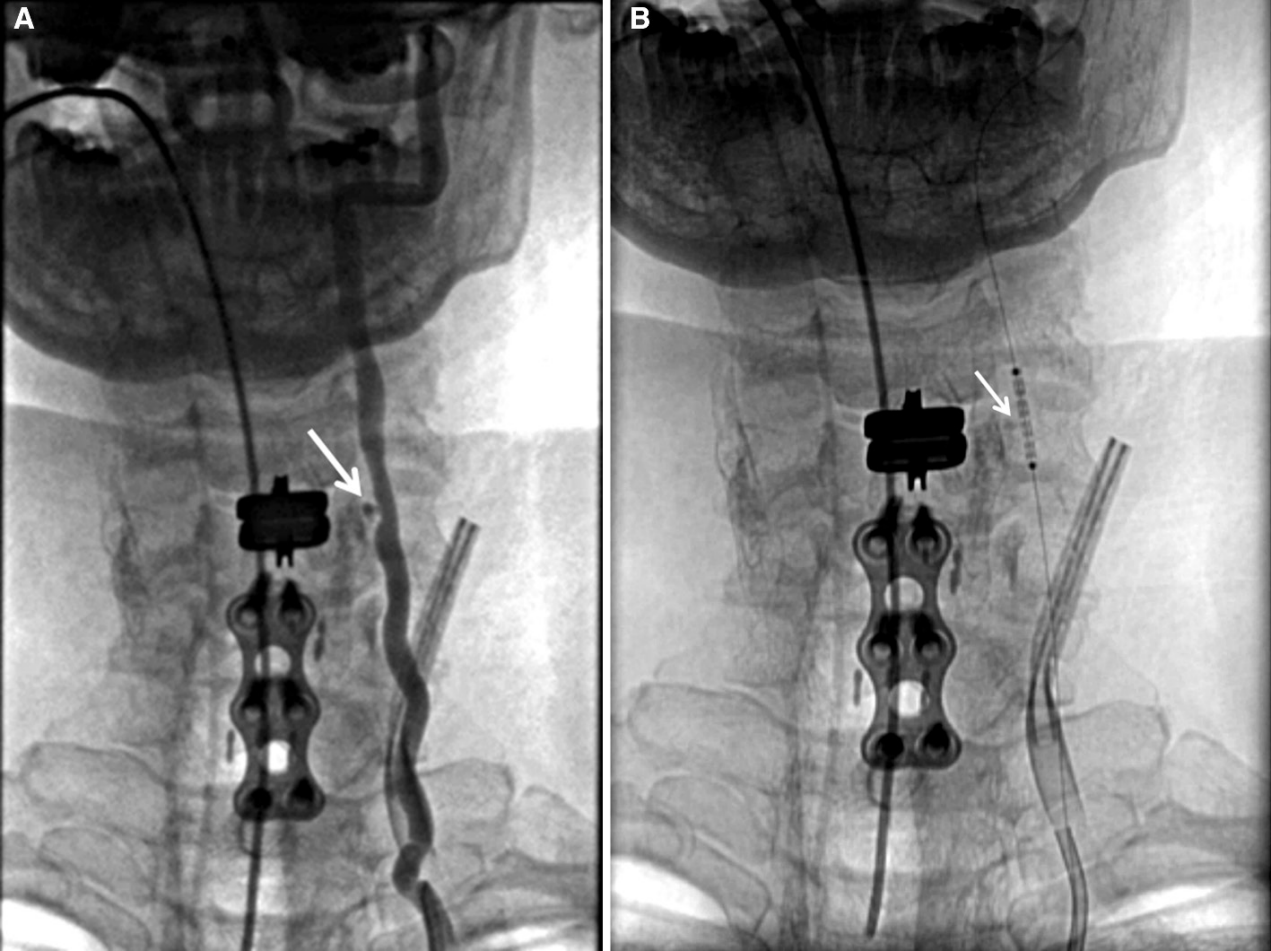
Left vertebral artery pseudoaneurysm. (a) Left vertebral artery angiography showing pseudoaneurysm in the V2 section between C5 and C6 (arrow). (b) Image showing a stent covered with ePTFE (arrow) before being deployed at C4–C5 level.

Angiography was performed with a JB1 catheter, with visualization of the right vertebral artery that ended in the posterior inferior cerebellar artery (PICA) and a pseudoaneurysm in the V2 segment of the left vertebral artery.(Figure 1) Anterior circulation was normal but the left posterior communicating artery was hypoplastic. Consequently, treatment with endovascular approach was attempted.

## Treatment

**Figure 2.**
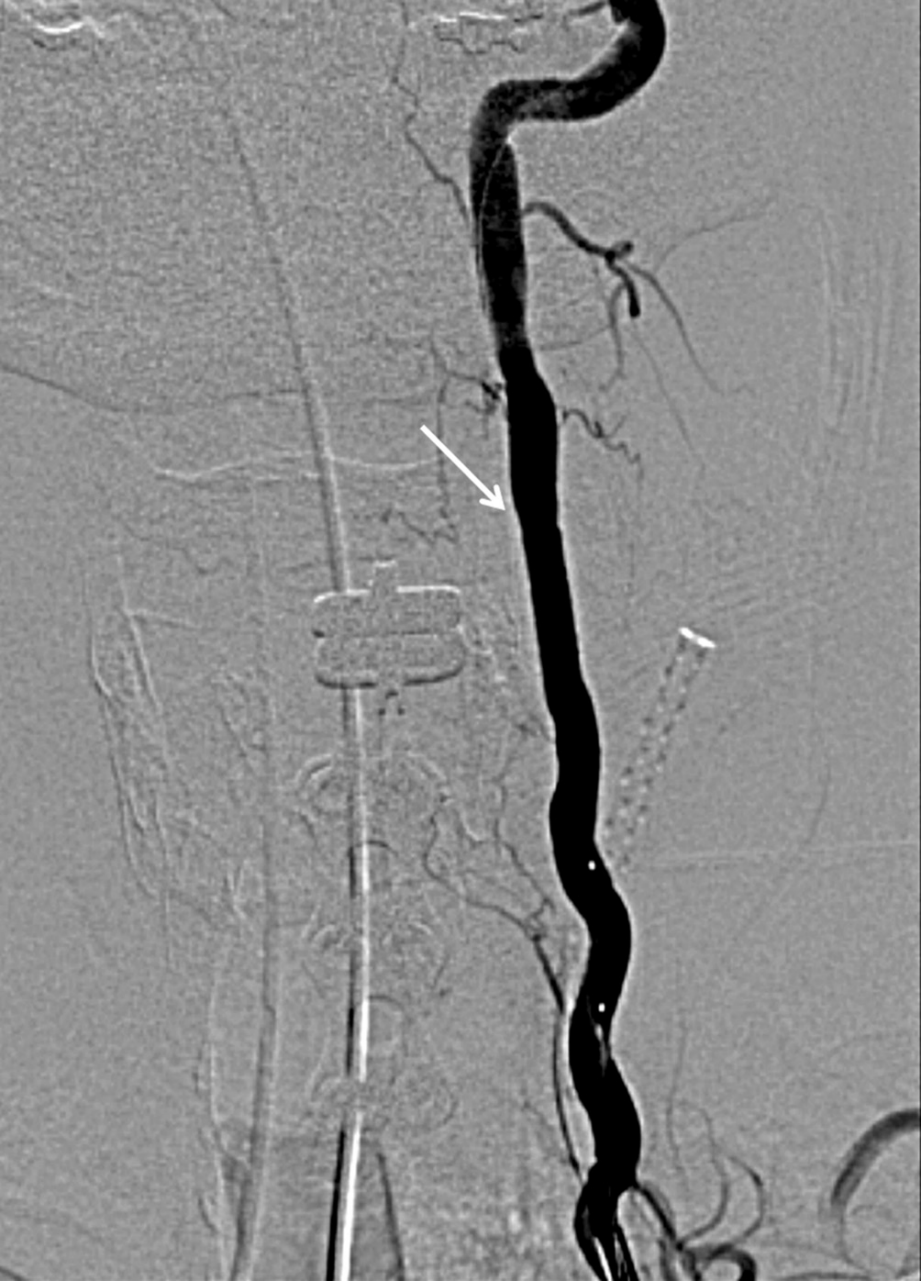
Left vertebral artery with stent. Left vertebral artery angiography after balloon expandable covered stent placement at C4–C5.

An i.v. bolus of 5000 IU of heparin was given and a multipurpose guide catheter was placed up to the left vertebral artery over a guidewire. The lesion was traversed with a 0.014 guidewire, and a 4 × 16 covered stent was easily guided towards the lesion and deployed. The post-deployment angiogram showed that the pseudoaneurysm was excluded from the circulation and no distal embolization was seen.(Figure 2)

## Outcome and follow-up

**Figure 3.**
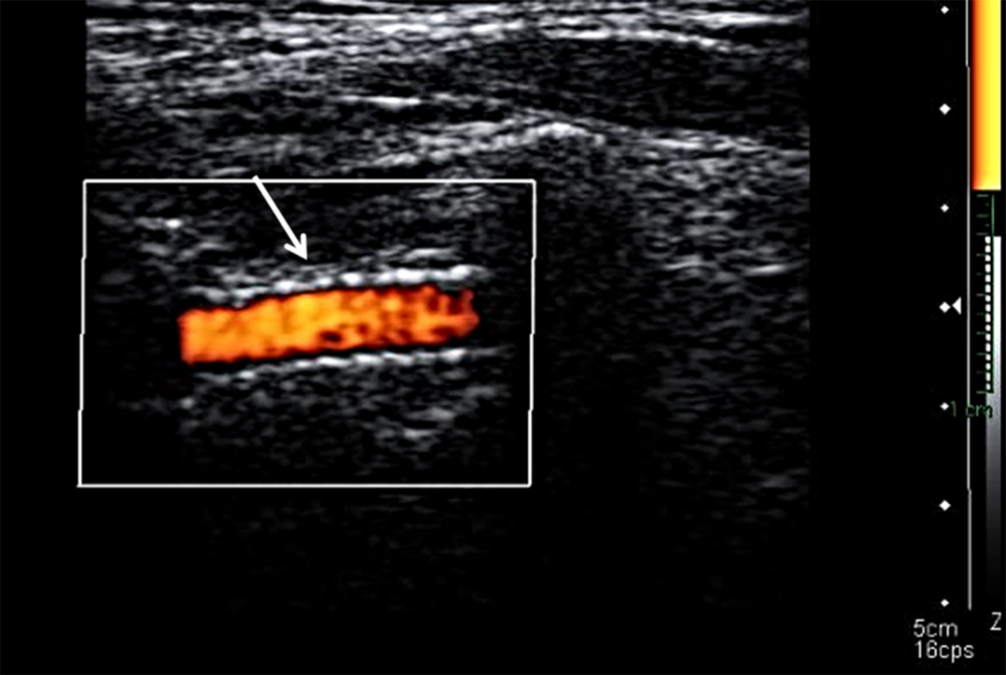
Left vertebral artery ultrasound. Doppler ultrasound performed during the 1-month follow-up visit. Ultrasound Doppler shows the implanted stent and normal flow inside.

During the postoperative period, the patient underwent surgical drainage of a cervical hematoma that developed during the initial surgery. The hematoma was displacing the trachea and the vascular structures of the neck. There were no neurological events after the procedures and the patient was discharged 5 days later. Medications after stent implantation consisted of aspirin and clopidogrel for 6 months, with initial anticoagulation with low molecular weight heparin during the first 5 days. Follow-up CT angiography and color Doppler ultrasound (Figure 3) performed a month later showed no in-stent stenosis and no evidence of infarction in the posterior circulation. The patient remained asymptomatic.

## Discussion

Pseudoaneurysm formation is the result of partial to complete disruption of the vascular wall, which ultimately leads to hemorrhage that is contained by the adventitia of the vessel wall or the perivascular soft tissues.^1^ Vertebral artery pseudoaneurysms are very rare and may be the result of cervical penetrating or blunt trauma, including any surgical procedure, arterial dissection, or diseases affecting collagen formation. The extradural or V3 segment of this artery is most prone to injury because of its path and position.^2^ In this case, the foraminal or V2 segment of the vertebral artery was affected due to fact that the surgical approach was done between vertebras C4 and C6.

Little is known about the progression of this type of aneurysm. However, manifestations may include the emergence of a local mass and neurologic symptoms related to disruptions in the posterior brain circulation such as limb and facial paralysis, ataxia, dysphagia, and hoarseness. Complications that may arise include rupture and thrombosis. Since signs and symptoms may appear long after its formation, imaging techniques provide an earlier diagnosis.^3^ Conventional angiography serves as the traditional imaging evaluation of patients suspected to have arterial injuries of the neck. However, the use of noninvasive imaging techniques for the assessment of vascular injuries is starting to become the standard. Artifacts caused by certain materials such as metal may influence the ability of CT angiography to uncover arterial injuries. This is why small pseudoaneurysms may not be fully disclosed and can be misdiagnosed with this technique.^1^ In these cases, angiography is the optimal method for assessment of vascular lesions.

Various sources agree that treatment of patients with vertebral artery pseudoaneurysms must be determined individually.^3^ A covered stent is probably the optimal treatment in terms of time and invasiveness, as it provides immediate sealing of the lesion and allows salvation of the vertebral artery.^4^ In the proper scenarios, deployment of covered stents can lead to exclusion of pseudoaneurysms with conservation of patency of the vessel. This should be the treatment of choice when the alternative surgical treatment is ligation of the injured artery.^4^ Anatomical variations in the Circle of Willis and the entire posterior circulation should be carefully evaluated. Aneurysms in the third and fourth portion of the artery are the most difficult to treat.^3^ In this case, the treated artery gave circulation to the posterior fossa as the right vertebral artery ended in PICA.

## Learning points

1. Vertebral artery pseudoaneurysm must be diagnosed and treated immediately because of its risk of rupture or thrombosis.
2. Angiography must be elected as the diagnostic method when artifacts caused by metal could be a problem. In other cases, CT angiography could be a reasonable alternative.
3. Endovascular covered stents' placement is an effective treatment method for the traumatically injured vertebral artery.

## References

[1] Núñez DB, Torres-León M, Múnera F Vascular injuries of the neck and thoracic inlet: helical CT–Angiographic correlation. RadioGraphics 2004; 24: 1087–98. doi: 10.1148/rg.244035035 15256630

[2] Ambekar S, Sharma M, Smith D, Cuellar H Successful treatment of iatrogenic vertebral pseudoaneurysm using pipeline embolization device. Case Rep Vasc Med 2014; 2014(1, article e4): 1–4. doi: 10.1155/2014/341748 PMC4167810 25276469

[3] Schittek A Pseudoaneurysm of the vertebral artery. Tex Heart Inst J 1999; 26: 90–5. 10217474 PMC325602

[4] Hüttl K, Sebestyén M, Entz L, Molnár AA, Nemes B, Bérczi V, et al Covered stent placement in a traumatically injured vertebral artery. J Vasc Interv Radiol 2004; 15(2 Pt 1): 201–2. doi: 10.1097/01.RVI.0000109406.52762.98 14963190

